# Efficacy of Chinese herbal medicine in the treatment of anxiety and depression in male sexual dysfunction: a systematic review and meta-analysis protocol

**DOI:** 10.3389/fpsyt.2025.1584306

**Published:** 2025-05-02

**Authors:** Zhaozhan Xie, Xuecheng Zhang, Hongling Jia, Yongchen Zhang

**Affiliations:** ^1^ School of Acupuncture-Moxibustion and Tuina, Shandong University of Chinese Medicine, Jinan, Shandong, China; ^2^ Department of Proctology, China-Japan Friendship Hospital, Beijing, China; ^3^ Department of Acupuncture and Moxibustion, Second Affiliated Hospital of Shandong University of Traditional Chinese Medicine, Jinan, Shandong, China

**Keywords:** male sexual dysfunction, chinese herbal medicine, anxiety, depression, systematic review, psychosexual health

## Abstract

**Background:**

Male Sexual Dysfunction (MSD), comprising erectile dysfunction (ED), premature ejaculation, and hypoactive sexual desire disorder, exhibits an age-related prevalence affecting 50% of males beyond their fourth decade. Epidemiological studies demonstrate ED prevalence rates of 79.4% in general clinical populations. Beyond physiological manifestations, MSD with comorbid anxiety and depression exerts profound psychosocial impacts, potentially exacerbating pre-existing mental health conditions and impairing interpersonal relationships. Emerging evidence suggests Chinese Herbal Medicine (CHM) may offer therapeutic potential for addressing this clinical intersection. Therefore, this study will perform a systematic review and meta-analysis to assess the efficacy of various CHM interventions for MSD patients with comorbid anxiety and depression.

**Methods:**

This study will systematically search four Chinese databases (China National Knowledge Infrastructure, Wanfang Database, China Biomedical Database, and VIP Database) and four international databases (PubMed, Web of Science, EMBASE, and Cochrane Library). Randomized controlled trials (RCTs) investigating CHM interventions for MSD with comorbid anxiety and depression will be identified. The retrieved studies will undergo rigorous screening and quality assessment using standardized tools.

**Results:**

The findings of this investigation will establish an evidence-based foundation for optimizing CHM therapeutic protocols in managing MSD with comorbid anxiety and depression.

**Conclusions:**

This investigation will synthesize evidence-based data to evaluate therapeutic outcomes of various CHM modalities for MSD with comorbid anxiety and depression. Through comparative effectiveness analyses, the study aims to establish hierarchical treatment recommendations, enabling clinicians to optimize intervention strategies and facilitate personalized treatment selection in clinical practice.

**Clinical trial registration:**

https://www.crd.york.ac.uk/prospero, identifier CRD420250652254

## 1 Introduction

Male Sexual Dysfunction (MSD) encompasses a range of conditions that impair male sexual function, including erectile dysfunction (ED), premature ejaculation (PE), and hypoactive sexual desire disorder (1). For men, ED and PE are the two most prevalent subtypes of male sexual dysfunction, with clinical presentations typically categorized as primary or secondary (2). ED is defined as the persistent inability of the penis to achieve or maintain an erection sufficient for satisfactory sexual activity. Based on etiological differences, ED can be categorized into three types: psychogenic ED, organic ED, and mixed ED (3, 4). PE can be defined as ejaculation that almost always occurs within approximately one minute of vaginal penetration since the first sexual intercourse (primary premature ejaculation), or a marked reduction in intravaginal latency time (IELT) to less than three minutes, typically due to a previous absence of such issues (secondary premature ejaculation) (5). The prevalence of MSD increases with age; approximately 50% of men over the age of 40 experience varying degrees of sexual dysfunction (6). The Massachusetts Male Aging Study (MMAS) reported that among men aged 40 to 70 in the Boston area, the overall prevalence of ED was 52%, with 50% to 60% of cases attributed to penile vascular dysfunction and 16% to 78% linked to mental health disorders (7–10).The overall incidence of PE is estimated to be between 20% and 30% (11), with 36% to 63% classified as primary premature ejaculation and 16% to 28% as secondary premature ejaculation (12, 13). Beyond its impact on physical health, MSD significantly affects mental well-being and interpersonal relationships, potentially exacerbating psychological issues such as anxiety and depression (14). The pathophysiological mechanisms underlying MSD are multifaceted, encompassing neurological, vascular, endocrine, and psychological factors. ED is commonly associated with inadequate penile blood flow, which may result from cardiovascular conditions such as atherosclerosis, diabetes mellitus, or hypertension (15). Endocrine dysregulation in patients with diabetes not only impairs testosterone synthesis but may also exacerbate ED by compromising vascular endothelial function (16). Research indicates that the prevalence of sexual dysfunction in individuals with anxiety disorders is significantly higher compared to the general population, and elevated anxiety levels are strongly associated with reduced sexual satisfaction (17). Furthermore, lifestyle factors such as smoking, excessive alcohol consumption, and physical inactivity can also adversely affect sexual function (18). Research indicates that sexual dysfunction is closely associated with anxiety and depression, forming a complex interrelationship that often traps patients in a vicious cycle. Anxiety and depression not only exacerbate the symptoms of sexual dysfunction but can also lead to avoidance of sexual activities, thereby further increasing the psychological burden on patients (19, 20). There is a significant association between depression and sexual dysfunction, particularly among male patients, where the prevalence of depression can reach up to 60% or higher (21, 22). Therefore, it is imperative to accord increased attention to anxiety and depression in the context of MSD.

Currently, the treatment options for MSD primarily include pharmacotherapy, psychotherapeutic interventions, and lifestyle modifications. In drug therapy, phosphodiesterase-5 inhibitors (PDE5Is) represent the first-line therapy for ED, while selective serotonin reuptake inhibitors (SSRIs) are primarily used for managing PE (23, 24). To date, four drugs have been approved by both the FDA and EMA: Sildenafil, Tadalafil, Vardenafil, and Avanafil. The therapeutic efficacy of these agents has been thoroughly validated. PDE5Is enhance NO-mediated vascular smooth muscle relaxation and promote increased blood flow and erection maintenance by inhibiting PDE5 enzyme activity and reducing cGMP degradation in the penile corpus cavernosum. Selective PDE5 inhibitors are effective agents for promoting penile erection, with their primary role being to support erection maintenance rather than directly induce erection onset (23). SSRIs are a class of antidepressants. Clinical observations have revealed their ability to prolong Intravaginal Ejaculation Latency Time (IELT), leading to their widespread use in the treatment of PE. Common SSRIs include dapoxetine hydrochloride, paroxetine, sertraline, and fluoxetine. These agents function by binding to and inhibiting 5-HT reuptake receptors on the presynaptic membrane, thereby increasing the concentration of 5-HT in the synaptic cleft. This process desensitizes 5-HT1A and 5-HT1B receptors, ultimately contributing to the prolongation of IELT and aiding in the management of PE (25, 26). It is worth noting that existing studies have explored the application of PDE5i in the treatment of PE, indicating that PDE5i may exert therapeutic effects through multiple mechanisms. These include alleviating sexual anxiety, reducing sympathetic nerve excitation, and promoting the dilation of smooth muscles in the vas deferens and seminal vesicles, all of which potentially contribute to delaying ejaculation (25, 27). However, while these medications can improve MSD symptoms to some extent, their efficacy in addressing concomitant psychological issues such as anxiety and depression is limited (28). It is worrying that common adverse reactions of SSRI drugs include fatigue, nausea, diarrhea and excessive sweating; while PDE5i drugs may cause discomfort such as headache, indigestion, vision problems and myalgia (26, 29). In addition, due to the high cost of treatment and limited efficacy, many patients eventually choose to discontinue treatment (23, 30). In psychological therapy interventions, a comprehensive analysis and evaluation of the psychological issues present in patients with ED and PE should be performed. By alleviating their inner fears and reducing anxiety and depressive symptoms, patients can be assisted in confronting their sexual dysfunction. The primary goal of psychological therapy is to eliminate fear, anxiety, and depression related to sexual activity while fostering effective communication between patients and their partners. Furthermore, sexual psychological therapy may be combined with behavioral techniques, such as the “start-stop” method and the “squeeze” technique (2, 31). The most critical aspect is the adjustment of lifestyle. Patients should conduct self-health management under medical supervision and maintain long-term treatment adherence. Specifically, establishing regular living habits and adhering to a consistent medication schedule are crucial for effective disease management. Treatment plans should be tailored as much as possible to individual patient needs. For instance, during the early stages of treatment or in cases of severe symptoms, a combination of multiple therapeutic approaches may be warranted (2, 23). Against this backdrop, Chinese Herbal Medicine (CHM) have increasingly garnered attention. Studies have demonstrated their efficacy in improving sexual function and alleviating anxiety and depression, with relatively fewer side effects compared to conventional treatments (16).

CHM has demonstrated significant advantages in treating anxiety and depression among patients with MSD, particularly in improving psychological well-being, enhancing sexual function, and providing robust clinical research evidence. The multi-component and multi-target nature of CHM confers a unique advantage in treating complex diseases, as its ability to simultaneously act on multiple pathological mechanisms enhances therapeutic efficacy (20, 32). Through data mining of medication patterns for male sexual dysfunction, it was revealed that Cistanche, Epimedium, and Lycium barbarum are frequently utilized in clinical practice (33, 34). Specifically, Cistanche enhances sexual function by elevating testosterone levels, exerting antioxidant properties, and combating hypoxia (35). Epimedium significantly boosts plasma testosterone levels and stimulates the proliferation and secretory functions of testicular tissue (36). Additionally, Lycium barbarum exhibits immunomodulatory effects, increases blood testosterone concentrations, and provides a tonic effect (37). Compound traditional Chinese medicine represents another effective therapeutic approach for male sexual dysfunction. Studies have demonstrated that Shugan Yiyang Capsules can significantly upregulate the gene and protein expression levels of the three nitric oxide synthase (NOS) subtypes, as well as enhance the protein expression of cyclic guanosine monophosphate (cGMP), ultimately contributing to the effective improvement of sexual dysfunction (38). CHM prescriptions, such as Bazhen Decoction and Sijunzi Decoction, demonstrate significant efficacy in regulating qi and blood and enhancing physical constitution, while also effectively alleviating symptoms of anxiety and depression, thereby contributing to improved sexual function (17, 39). However, although individual studies have explored the effects of CHM on MSD and associated psychological conditions including anxiety and depression, the research findings remain inconsistent. Particularly in cases involving comorbidities between MSD and mental health disorders such as anxiety and depression, a comprehensive evaluation of the therapeutic efficacy of CHM remains to be established.

Consequently, the goal of this proposed meta-analysis is to address the existing knowledge gap regarding CHM in managing MSD with comorbid anxiety and depression. By systematically synthesizing evidence from clinical studies, we aim to evaluate CHM’s therapeutic potential and provide a foundational framework for future randomized controlled trials (RCTs), ultimately supporting evidence-based clinical decisions

## 2 Materials and methods

This study will adhere to the Preferred Reporting Items for Systematic Reviews and Meta-Analyses (PRISMA) guidelines (40, 41). The registration number of the protocol is CRD420250652254 (https://www.crd.york.ac.uk/prospero/).

### 2.1 Search strategy

Two researchers comprehensively searched 4 Chinese databases, including China National Knowledge Infrastructure (CNKI), Wanfang Data Knowledge Service Platform, VIP, and CBM, and 4 English databases, including PubMed, Web of Science, EMBASE, and The Cochrane Library. We searched the database for all articles from its inception to 24 February 2025 in Chinese and English, with no geographical restrictions. A combination of subject matter and free terminology is employed to ensure a comprehensive search, regardless of language or type of publication. All databases will be searched to ensure that all relevant articles are identified. The detailed search strategy for the PubMed database is presented in **Table 1**.

**Table 1:** The search strategy of Pubmed.

Search step	Search query
#1	male sexual dysfunction[Mesh] OR erectile dysfunction[Title/Abstract] OR premature ejaculation[Title/Abstract]
#2	anxiety OR depression
#3	Chinese herbal medicine OR traditional Chinese medicine
#4	randomized controlled trial[Publication Type] OR randomized[Title/Abstract] OR placebo[Title/Abstract]
#5	#1 AND #2 AND #3 AND #4

### 2.2 Inclusion and exclusion criteria

#### 2.2.1 Type of study

Only RCTs were included. However, the studies must be in either English or Chinese. Observational studies, cross-sectional studies, animal studies, and other non-RCT designs were excluded because they are highly susceptible to confounding factors that may compromise the causal inference of CHM efficacy.

#### 2.2.2 Type of participants

Adult male patients diagnosed with MSD and comorbid anxiety and/or depression.

#### 2.2.3 Type of intervention

The experimental group must receive treatment with either single-agent or combination CHM, while the control group may receive placebo, conventional pharmacotherapy, or no intervention.

#### 2.2.4 Type of outcome measures

Outcome measures required at least one assessment of anxiety, depression, or overall symptoms. Primary outcomes included the Self-Rating Anxiety Scale (SAS) (42), the Self-Rating Depression Scale (SDS) (43), the Hamilton Anxiety Rating Scale (HAMA) (44), the Hamilton Depression Rating Scale (HAMD) (45), and the Hospital Anxiety and Depression Scale (HADS) (46). Secondary outcomes pertained to the global alleviation of symptoms (47).

### 2.5 Study selection process and data extraction

**Figure 1:**
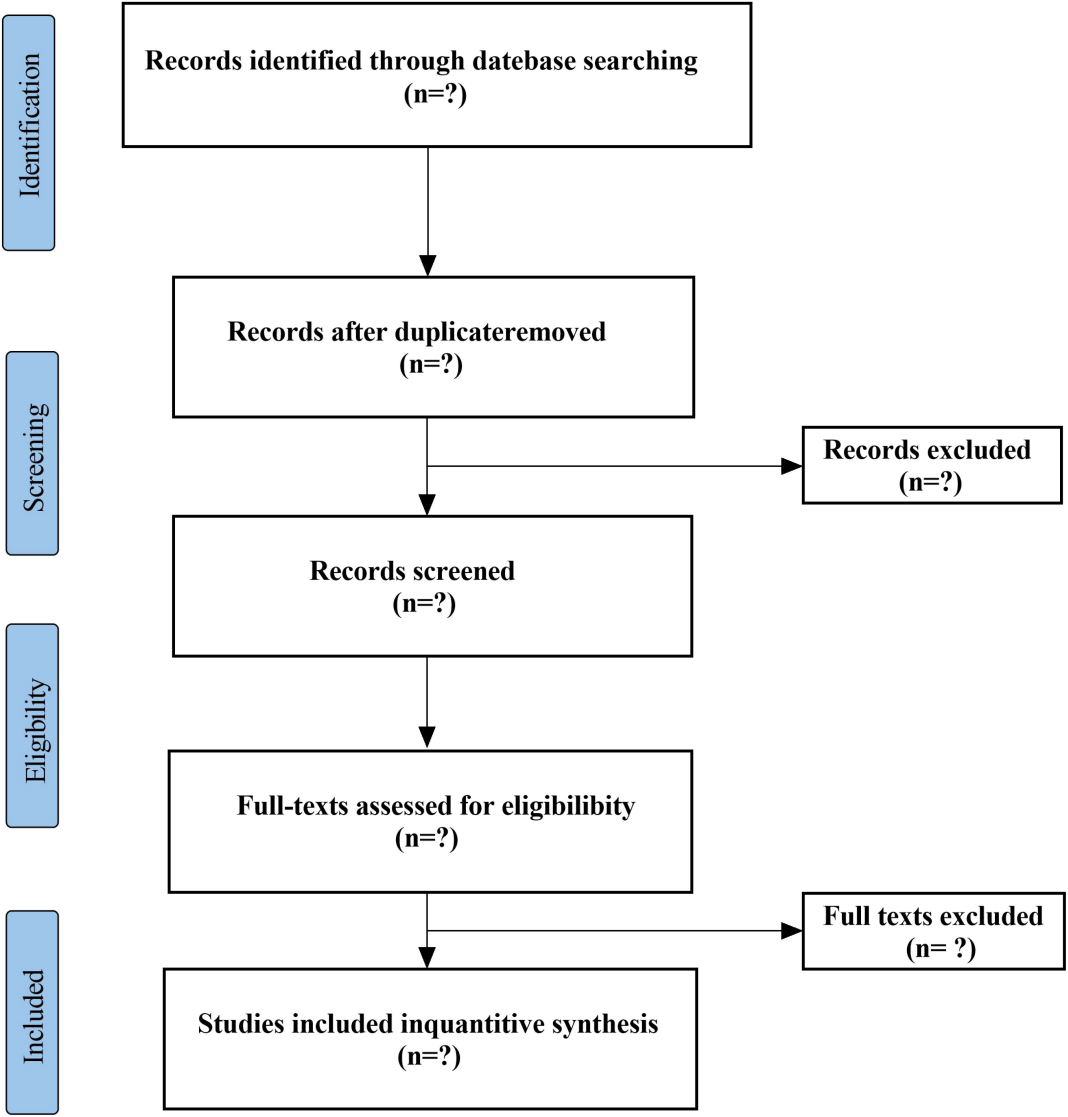
PRISMA flow chart.

Two researchers will independently screen titles and abstracts to confirm whether they meet the inclusion criteria, and will conduct full-text evaluations of all included studies. The following data will be extracted using standardized forms: first author’s name, year of publication, age range of participants, sample size, diagnostic criteria, interventions in the treatment and control groups, duration of treatment, outcome measures, and adverse events. In the event of disagreements, a third researcher will be consulted, and a final decision will be made through discussion. The PRISMA flowchart (**Figure 1**) will provide a comprehensive overview of the selection process.

### 2.6 Risk of bias assessment

Two researchers will independently assess the risk of bias for each included randomized controlled trial (RCT) using the Cochrane Handbook-recommended Risk of Bias tool, RoB 2.0 (48). The assessment will cover the following domains: random sequence generation, allocation concealment, blinding of participants and personnel, blinding of outcome assessors, incomplete outcome data, selective reporting, and other potential sources of bias. Based on the assessment, each study will be classified as having a high, low, or unclear risk of bias. In the event of disagreements, a third researcher will be consulted to assist in reaching consensus.

### 2.7 Statistical analysis

We will perform a meta-analysis using STATA. As each score represents a numeric variable with potentially different measurement scales and scoring criteria, we will extract score differences before and after treatment. The standardized mean difference (SMD) will be selected as the effect measure, with 95% confidence intervals (95% CI) serving as the statistical test intervals for effect estimates. Heterogeneity will be evaluated using the Chi-squared test and I² statistic. If studies demonstrate low heterogeneity (I² < 50%), we will employ a fixed-effect model for meta-analysis. For substantial heterogeneity (I² ≥ 50%), a random-effects model will be utilized, followed by subgroup analyses to investigate heterogeneity sources. When quantitative synthesis is inappropriate, we will provide narrative synthesis of results for publication inclusion. For trials reporting only pre-post values, mean changes will be calculated by subtracting baseline measurements from post-intervention values, with corresponding standard deviations (SD) of change estimated accordingly.

### 2.8 Subgroup analyses

In cases of significant heterogeneity, planned subgroup analyses will be conducted to explore potential sources of heterogeneity by examining the following factors: Age (<40 vs. ≥40 years), Baseline depression severity (mild [PHQ-9 <10] vs. moderate-severe [PHQ-9 ≥10]), CHM intervention type (single-herb vs. compound formulations; short-term [<8 weeks] vs. long-term [≥8 weeks] regimens). When adequate data exists across subgroups, we will perform quantitative analyses using mixed-effects models to examine subgroup-by-treatment interactions. For subgroups with insufficient data for quantitative synthesis, a structured qualitative synthesis will be implemented, involving within-study comparisons, cross-study pattern analysis, and evidence grading through GRADE criteria, enabling meaningful interpretation without formal meta-analytic combination (49).

### 2.9 Sensitivity analysis

Should significant heterogeneity persist following subgroup analyses, we will conduct sensitivity analyses to evaluate result stability. The sensitivity analysis protocol will involve systematically re-running the meta-analysis while excluding studies with high risk of bias (RoB ≥ 4 on modified Newcastle-Ottawa Scale) and statistical outliers identified through Galbraith plots. By comparing effect size estimates between primary and sensitivity analyses using Hartung-Knapp adjustment, we will quantify the influence of individual studies on pooled effects. This methodological approach enables assessment of result robustness while clarifying potential heterogeneity drivers through differential exclusion impacts.

### 2.10 Publication bias

When the meta-analysis includes 10 or more studies, we will evaluate publication bias using Egger’s regression test. The results will be visually complemented with funnel plots to assess potential asymmetry in effect size distribution.

## 3 Discussion

In recent years, research on the use of traditional CHM for managing anxiety and depression symptoms associated with MSD has garnered increasing attention. CHM demonstrates potential advantages in simultaneously addressing sexual dysfunction and psychological comorbidities through its multi-target mechanisms, contrasting with conventional therapies limited to isolated symptom management. Although systematic reviews inherently rely on existing data rather than recruiting new patients, the publication of a protocol remains methodologically essential. First, pre-registration reduces the risk of unintended deviations from predefined inclusion criteria and statistical methods, thereby minimizing selective reporting bias (41). Second, given that CHM trials often exhibit heterogeneous outcome measures, publishing the protocol in advance ensures transparency in standardizing these endpoints. Finally, considering the ongoing debate regarding methodological standards for CHM reviews (50), this protocol adheres to PRISMA-P guidelines to establish a rigorous precedent for evidence synthesis in integrative psychosexual medicine.

CHM exerts therapeutic effects on MSD with comorbid anxiety and depression through multi-target interventions involving neurotransmitter regulation, metabolic modulation, and neuroendocrine-immune integration. CHM formulations restore serotonin (5-HT) and norepinephrine balance by inhibiting monoamine oxidase activity and upregulating synaptic neurotransmitter reuptake. For instance, Chaihu Shugan San, which contains Saikosaponins, has been shown to significantly improve HAMD scores via this mechanism (51–53). Herbs such as Gardenia jasminoides, which contain iridoids, suppress corticosterone overproduction and downregulate glucocorticoid receptor expression in hippocampal neurons, thereby reversing stress-induced dysregulation of the HPA axis (54). Saikosaponins (SSs), the primary active components of Bupleurum scorzonerifolium Willd, have demonstrated significant improvement in depression-like behavior, attenuation of central inflammation, and marked inhibition of neuronal pyroptosis in mice across both *in vivo* and *in vitro* models (55). CHM formulas suppress hyperactivity of the hypothalamic-pituitary-adrenal (HPA) axis and reduce proinflammatory cytokines by modulating glucocorticoid receptor expression. For instance, iridoids from Gardenia jasminoides exert anti-inflammatory effects through NF-κB pathway inhibition, thereby restoring neuroendocrine-immune homeostasis (56). Tianmeng Oral Liquid alleviate depressive symptoms by modulating nucleotide, energy, and amino acid metabolism, thereby replenishing ATP and reducing oxidative stress in MSD-related neuronal circuits (57). The multicomponent nature of CHM enables it to simultaneously target neurotransmitter imbalance, inflammation, and vascular dysfunction, thereby distinguishing it from conventional single-pathway antidepressants (56, 58).

However, in the research and clinical application of traditional CHM, several challenges also arise. Firstly, the scientific evidence supporting the efficacy and mechanisms of action of CHM remains relatively limited. While some preliminary studies have demonstrated promising results, large-scale, RCTs are still scarce. Secondly, due to the complex composition of CHM, standardizing dosage and usage methods is critically important. This lack of standardization may compromise the reproducibility and safety of therapeutic outcomes.Therefore, it is of great significance to systematically evaluate the efficacy and safety of CHM in treating MSD accompanied by anxiety and depression, and to explore its integration with traditional treatment regimens.

We will analyze published RCTs to comprehensively assess the therapeutic effects of various CHM formulations, with the aim of identifying optimal administration methods, treatment duration, and dosage regimens. The findings are anticipated to provide evidence-based guidance for clinical practice and establish a theoretical foundation for integrating CHM into comprehensive treatment strategies for sexual dysfunction with psychological comorbidities. However, this systematic review and network meta-analysis may encounter several methodological limitations, including potential publication bias, clinical heterogeneity, and selection bias. Firstly, the predetermined stringent inclusion criteria, although crucial for ensuring methodological rigor, might limit the number of eligible RCTs. Future reviews could consider incorporating high-quality observational studies to investigate the long-term safety and adherence patterns of CHM in real-world settings. Secondly, linguistic restrictions limited to English and Chinese publications could introduce geographical selection bias. Finally, we will focus on the most widely used and representative CHM formulations, which may limit the exploration of less common but potentially effective therapies. Despite these limitations, we anticipate that this study will establish an evidence base for CHM in managing MSD with comorbid anxiety and depression, while offering novel perspectives for psychosexual integrative care.

## References

[1] Association AP . Diagnostic and Statistical Manual Of Mental Disorders. 5th ed. The United States: American Psychiatric Association Publishing (2013) p. 423–50.

[2] Expert Consensus Writing Group of Branch of Sexology of Traditional Chinese Medicine CSA . Erectile dysfunction and premature ejaculation comorbidity diagnosis and treatment by integrated Traditional Chinese and Western Medicine Chinese Expert Consensus. Chin J Exper Tradit Med Form. (2024) 30:147–53. doi: 10.13422/j.cnki.syfjx.20241093

[3] Minhas S Bettocchi C Boeri L Capogrosso P Carvalho J Cilesiz NC . European association of urology guidelines on male sexual and reproductive health: 2021 update on male infertility. Eur Urol. (2021) 80:603–20. doi: 10.1016/j.eururo.2021.08.014 34511305

[4] Salonia A Bettocchi C Boeri L Capogrosso P Carvalho J Cilesiz NC . European association of urology guidelines on sexual and reproductive health-2021 update: male sexual dysfunction. Eur Urol. (2021) 80:333–57. doi: 10.1016/j.eururo.2021.06.007 34183196

[5] Althof SE Mcmahon CG Waldinger MD Serefoglu EC Shindel AW Adaikan PG . An update of the international society of sexual medicine’s guidelines for the diagnosis and treatment of premature ejaculation (PE). Sex Med. (2014) 2:60–90. doi: 10.1002/sm2.28 25356302 PMC4184677

[6] Anderson D Laforge J Ross MM Vanlangendonck R Hasoon J Viswanath O . Male sexual dysfunction. Health Psychol Res. (2022) 10:37533. doi: 10.52965/001c.37533 35999971 PMC9392840

[7] Dong W Niu H . Application of penile vascular reconstruction in the treatment of vascular erectile dysfunction. Chin J Hum Sex. (2015) 24:41–3. doi: 10.3969/j.issn.1672-1993.2015.02.014

[8] Feldman HA Goldstein I Hatzichristou DG Krane RJ Mckinlay JB . Impotence and its medical and psychosocial correlates: results of the massachusetts male aging study. J Urol. (1994) 151:54–61. doi: 10.1016/s0022-5347(17)34871-1 8254833

[9] Hatzichristou DG Hatzimouratidis K Ioannides E Yannakoyorgos K Dimitriadis G Kalinderis A . Nocturnal penile tumescence and rigidity monitoring in young potent volunteers: reproducibility, evaluation criteria and the effect of sexual intercourse. J Urol. (1998) 159:1921–6. doi: 10.1016/S0022-5347(01)63197-5 9598488

[10] Lue TF Giuliano F Montorsi F Rosen RC Andersson K Althof S . Summary of the recommendations on sexual dysfunctions in men. J Sex Med. (2004) 1:6–23. doi: 10.1111/j.1743-6109.2004.10104.x 16422979

[11] Gao J Peng D Zhang X Hao Z Zhou J Fan S . Prevalence and associated factors of premature ejaculation in the anhui male population in China: evidence-based unified definition of lifelong and acquired premature ejaculation. Sex Med. (2017) 5:e37–43. doi: 10.1016/j.esxm.2016.11.002 PMC5302376 28041923

[12] Zhang X Gao J Liu J Xia L Yang J Hao Z . Distribution and factors associated with four premature ejaculation syndromes in outpatients complaining of ejaculating prematurely. J Sex Med. (2013) 10:1603–11. doi: 10.1111/jsm.12123 23534914

[13] Serefoglu EC Cimen HI Atmaca AF Balbay MD . The distribution of patients who seek treatment for the complaint of ejaculating prematurely according to the four premature ejaculation syndromes. J Sex Med. (2010) 7:810–5. doi: 10.1111/j.1743-6109.2009.01570.x 19912501

[14] Pitta RM Kaufmann O Luz JSN Ritti-Dias RM Queiroga LDL Wolosker N . The association between erectile dysfunction and depression: a cross-sectional study of 21,139 Brazilian men. Einstein (Sao Paulo). (2024) 22:eAO1063. doi: 10.31744/einstein_journal/2024AO1063 39699403 PMC11634340

[15] Flegge LG Barr A Craner JR . Sexual functioning among adults with chronic pain: prevalence and association with pain-related outcomes. Pain Med. (2023) 24:197–206. doi: 10.1093/pm/pnac117 35929084

[16] Liu Y Jin B . Mechanism of traditional chinese medicine extract in the treatment of diabetic erectile dysfunction. J Ethnopharmacol. (2025) 341:119332. doi: 10.1016/j.jep.2025.119332 39778785

[17] Strizzi JM Hald GM Pavan S Heymann-Szlachcinska A Ollgaard M Winding C . Predictors of sexual dysfunction, associated distress, and sexual satisfaction among male and female patients living with anxiety disorders in Denmark. J Sex Res. (2024), 1–16. doi: 10.1080/00224499.2024.2432608 39670954

[18] Jia B Li Z Zhao D Fu Q . Research progress on ferroptosis in organic erectile dysfunction. Arch Esp Urol. (2023) 76:746–54. doi: 10.56434/j.arch.esp.urol.20237610.90 38186067

[19] Wang YL Geng LG He CB Yuan SY . Chinese herbal medicine combined with tadalafil for erectile dysfunction: a systematic review and meta-analysis. Andrology. (2020) 8:268–76. doi: 10.1111/andr.12696 31464074

[20] Wu H Gao Z Dai D Liu X Fang Y Chen X . Efficacy and safety assessment of traditional chinese medicine for erectile dysfunction: a meta-analysis and trial sequential analysis. Andrology. (2023) 11:1345–67. doi: 10.1111/andr.13420 36848898

[21] Sibley AA Shrestha S Lipovac-Dew M Kunik ME . Examining depression symptoms with/without coexisting anxiety symptoms in community-dwelling persons with dementia. Am J Alzheimers Dis Other Demen. (2021) 36:1312416379. doi: 10.1177/1533317521990267 PMC10624070 33530695

[22] Zhang W Nan N He Y Zuo H Song X Zhang M . Prevalence of depression and anxiety symptoms and their associations with cardiovascular risk factors in coronary patients. Psychol Health Med. (2023) 28:1275–87. doi: 10.1080/13548506.2022.2104885 35880259

[23] Longoni M Bertini A Schifano N Zaffuto E Maggio P Piercarlo R . A review on pharmacological options for the treatment of erectile dysfunction: state of the art and new strategies. Expert Opin Pharmacother. (2023) 24:1375–86. doi: 10.1080/14656566.2023.2221785 37272398

[24] Liu K Wang Z Liu Y Zhu P Zhang S Lu S . An electrophysiological technique to accurately diagnose and treat erectile dysfunction. J Vis Exp. (2022) 189:14. doi: 10.3791/63851 36408982

[25] Mitsogiannis I Dellis A Papatsoris A Moussa M . An up-to-date overview of the pharmacotherapeutic options for premature ejaculation. Expert Opin Pharmacother. (2022) 23:1043–50. doi: 10.1080/14656566.2022.2035361 35108136

[26] Waldinger MD . Drug treatment options for premature ejaculation. Expert Opin Pharmacother. (2018) 19:1077–85. doi: 10.1080/14656566.2018.1494725 30028639

[27] Abu El-Hamd M . Efficacy and safety of daily use of tadalafil in treatment of patients with premature ejaculation: a randomised placebo-controlled clinical trial. Andrologia. (2018) 50:e13005. doi: 10.1111/and.13005 29527702

[28] Shahrajabian MH . Powerful stress relieving medicinal plants for anger, anxiety, depression, and stress during global pandemic. Recent Pat Biotechnol. (2022) 16:284–310. doi: 10.2174/1872208316666220321102216 35319401

[29] Carvalheira AA Pereira NM Maroco J Forjaz V . Dropout in the treatment of erectile dysfunction with pde5: a study on predictors and a qualitative analysis of reasons for discontinuation. J Sex Med. (2012) 9:2361–9. doi: 10.1111/j.1743-6109.2012.02787.x 22616766

[30] Kim S Lee Y Seo K Jung G Kim T . Reasons and predictive factors for discontinuation of pde-5 inhibitors despite successful intercourse in erectile dysfunction patients. Int J Impot Res. (2014) 26:87–93. doi: 10.1038/ijir.2013.41 24305610 PMC4019980

[31] Zhang Y Guo J Zhang C Zhang G Yan S Chen L . Expert consensus on doctor-patient communication in the diagnosis and treatment of erectile dysfunction combined with premature ejaculation. Chin J Androl. (2016) 30:58–62. doi: 10.3969/j.issn.1008-0848.2016.09.014

[32] Xu R Ma J Zhang X Liao Z Fu Y Lv B . Efficacy of Chinese herbal medicine formula in the treatment of mild to moderate erectile dysfunction: study protocol for a multi-center, randomized, double-blinded, placebo-controlled clinical trial. Int J Gen Med. (2023) 16:5501–13. doi: 10.2147/IJGM.S436347 PMC10683653 38034900

[33] Yang Y Weng J Wu Q Zhao J Zhu H Cui C . Analysis of administration rules of chinese patent medicine for impotence based on data mining. New Chin Med. (2023) 55:36–42. doi: 10.13457/j.cnki.jncm.2023.07.006

[34] Shi X Xu H . Research on medication regularities in the premature ejaculation treatment based on data mining from “prepared traditional Chinese medicine formulas. Chin hea car. (2025) 43:27–31. Available online at: https://kns.cnki.net/kcms2/article/abstract?v=KlmjsyJjhUT8GxKcEnoYIHvDz-q6JjZGw3ce7TC74xABQMjnMoMAMJmmMrxJ2EKEzkXv1DO8yy2XmqNorg6uXs9r_hGnWcwUdsCGE7Y_S0hANun0KKhWLF4p-pYk0CqOqwcGWJ3KkIIklrYDHOHJDiXNC5L64ph5JentPjC-YOqxWmvJnvf9YPf_60yLnqw1WuJTk2Zo5YQ=&uniplatform=NZKPT&language=CHS.

[35] Li Z Li J Li Y Guo L Xu P Du H . The role of cistanches herba and its ingredients in improving reproductive outcomes: a comprehensive review. Phytomedicine. (2024) 129:155681. doi: 10.1016/j.phymed.2024.155681 38718638

[36] Szabo R Racz CP Dulf FV . Bioavailability improvement strategies for icariin and its derivates: a review. Int J Mol Sci. (2022) 23:12–7. doi: 10.3390/ijms23147519 PMC9318307 35886867

[37] Zhang M Yue K Jiang J Chen H . Research progress on pharmacological effects of lycii fructus and its active ingredients. Drug Eval Res. (2023) 46:1611–9. doi: 10.7501/j.issn.1674-6376.2023.07.027

[38] Wang J Wang Q Liu B Li D Yuan Z Zhang H . A Chinese herbal formula, shuganyiyang capsule, improves erectile function in male rats by modulating Nos-CGMP mediators. Urology. (2012) 79:241. doi: 10.1016/j.urology.2011.08.026 22070893

[39] Feng Y Shi T Fu Y Lv B . Traditional Chinese medicine to prevent and treat diabetic erectile dysfunction. Front Pharmacol. (2022) 13:956173. doi: 10.3389/fphar.2022.956173 36210810 PMC9532934

[40] Page MJ Moher D Bossuyt PM Boutron I Hoffmann TC Mulrow CD . PRISMA 2020 explanation and elaboration: updated guidance and exemplars for reporting systematic reviews. BMJ. (2021) 372:n160. doi: 10.1136/bmj.n160 33781993 PMC8005925

[41] Page MJ Mckenzie JE Bossuyt PM Boutron I Hoffmann TC Mulrow CD . The prisma 2020 statement: an updated guideline for reporting systematic reviews. BMJ. (2021) 372:n71. doi: 10.1136/bmj.n71 33782057 PMC8005924

[42] Zung WW . A rating instrument for anxiety disorders. Psychosomatics. (1971) 12:371–9. doi: 10.1016/S0033-3182(71)71479-0 5172928

[43] Zung WW . A self-rating depression scale. Arch Gen Psychiatry. (1965) 12:63–70. doi: 10.1001/archpsyc.1965.01720310065008 14221692

[44] Hamilton M . The assessment of anxiety states by rating. Br J Med Psychol. (1959) 32:50–5. doi: 10.1111/j.2044-8341.1959.tb00467.x 13638508

[45] Hamilton M . A rating scale for depression. J Neurol Neurosurg Psychiatry. (1960) 23:56–62. doi: 10.1136/jnnp.23.1.56 14399272 PMC495331

[46] Zigmond AS Snaith RP . The hospital anxiety and depression scale. Acta Psychiatr Scand. (1983) 67:361–70. doi: 10.1111/j.1600-0447.1983.tb09716.x 6880820

[47] Xiaoyu Z . Guidelines for Clinical Research of New Chinese Medicines (Trial). 05 ed. Beijing: China Medical Science and Technology Press (2002). p. 402.

[48] Sterne JAC Savovic J Page MJ Elbers RG Blencowe NS Boutron I . RoB 2: a revised tool for assessing risk of bias in randomised trials. BMJ. (2019) 366:l4898. doi: 10.1136/bmj.l4898 31462531

[49] Balshem H Helfand M Schunemann HJ Oxman AD Kunz R Brozek J . Grade guidelines: 3. Rating the quality of evidence. J Clin Epidemiol. (2011) 64:401–6. doi: 10.1016/j.jclinepi.2010.07.015 21208779

[50] Luo S Long Y Xiao W Wang X Chen R Guo Q . Risk of bias assessments and reporting quality of systematic reviews and randomized controlled trials examining acupuncture for depression: an overview and meta-epidemiology study. J Evid Based Med. (2020) 13:25–33. doi: 10.1111/jebm.12372 32112515

[51] Yang L Di YM Shergis JL Li Y Zhang AL Lu C . A systematic review of acupuncture and chinese herbal medicine for postpartum depression. Complement Ther Clin Pract. (2018) 33:85–92. doi: 10.1016/j.ctcp.2018.08.006 30396632

[52] Feng D Tang T Lin X Yang Z Yang S Xia Z . Nine Traditional Chinese herbal formulas for the treatment of depression: an ethnopharmacology, phytochemistry, and pharmacology review. Neuropsychiatr Dis Treat. (2016) 12:2387–402. doi: 10.2147/NDT.S114560 PMC5036551 27703356

[53] Li D Li X Duan J Cai W . Wuling capsule promotes hippocampal neurogenesis by improving expression of connexin 43 in rats exposed to chronic unpredictable mild stress. Zhong Xi Yi Jie He Xue Bao. (2010) 8:662–9. doi: 10.3736/jcim20100710 20619143

[54] Sun G Shih J Chiou S Hong C Lu S Pao L . Chinese herbal medicines promote hippocampal neuroproliferation, reduce stress hormone levels, inhibit apoptosis, and improve behavior in chronically stressed mice. J Ethnopharmacol. (2016) 193:159–68. doi: 10.1016/j.jep.2016.07.025 27416803

[55] Bi Y Li M Wang Y Yao J Wang Y Wang S . Saikosaponins from bupleurum scorzonerifolium willd. Alleviates microglial pyroptosis in depression by binding and inhibiting P2x7 expression. Phytomedicine. (2025) 136:156240. doi: 10.1016/j.phymed.2024.156240 39637473

[56] Li C Huang B Zhang Y . Chinese herbal medicine for the treatment of depression: effects on the neuroendocrine-immune network. Pharmaceuticals (Basel). (2021) 14:65. doi: 10.3390/ph14010065 33466877 PMC7830381

[57] Xie Z Zhang Z Xie C Guo Y . Sexual function of depression in men with combination of tianmeng oral liquid and anti-depressant. Chin Tradit Her Dru. (2018) 49:2620–3. doi: 10.7501/j.issn.0253-2670.2018.11.020

[58] Kang D Dong H Shen Y Ou J Zhao J . The clinical application of Chinese herbal medication to depression: a narrative review. Front Public Health. (2023) 11:1120683. doi: 10.3389/fpubh.2023.1120683 36969689 PMC10034025

